# Inhibition of MAPK‐Erk pathway in vivo attenuates aortic valve disease processes in Emilin1‐deficient mouse model

**DOI:** 10.14814/phy2.13152

**Published:** 2017-03-07

**Authors:** Charu Munjal, Anil G. Jegga, Amy M. Opoka, Ivan Stoilov, Russell A. Norris, Craig J. Thomas, J. Michael Smith, Robert P. Mecham, Giorgio M. Bressan, Robert B. Hinton

**Affiliations:** ^1^ Divisions of CardiologyCincinnati Children's Hospital Medical CenterCincinnatiOhio; ^2^ Biomedical InformaticsCincinnati Children's Hospital Medical CenterCincinnatiOhio; ^3^ Department of Cell Biology and PhysiologyWashington University School of MedicineSt. LouisOhio; ^4^ Department of Cell BiologyMedical University of South CarolinaCharlestonSouth Carolina; ^5^ Division of Pre‐Clinical InnovationNational Center for Advancing Translational SciencesNational Institutes of HealthBethesdaMaryland; ^6^ TriHealth Heart InstituteCardio‐Thoracic SurgeryCincinnatiOhio; ^7^ The Department of Biomedical SciencesUniversity of PaduaPaduaItaly

**Keywords:** Angiogenesis, elastases, elastic fibers, fibrosis, inflammation, valves

## Abstract

Aortic valve disease (AVD) is a common condition with a progressive natural history, and presently, there are no pharmacologic treatment strategies. Elastic fiber fragmentation (EFF) is a hallmark of AVD, and increasing evidence implicates developmental elastic fiber assembly defects. Emilin1 is a glycoprotein necessary for elastic fiber assembly that is present in both developing and mature human and mouse aortic valves. The *Emilin1‐*deficient mouse (*Emilin1* ^*−/−*^) is a model of latent AVD, characterized by activated TGFβ/MEK/p‐Erk signaling and upregulated elastase activity. *Emilin1* ^*−/−*^ aortic valves demonstrate early EFF and aberrant angiogenesis followed by late neovascularization and fibrosis. The objective of this study was to test the effectiveness of three different targeted therapies. Aged (12–14 months) *Emilin1* ^*−/−*^ mice were treated with refametinib (RDEA‐119, MEK1/2 inhibitor), doxycycline (elastase inhibitor), or G6‐31 (anti‐VEGF‐A mouse antibody) for 4 weeks. Refametinib‐ and doxycycline‐treated *Emilin1* ^*−/−*^ mice markedly reduced MEK/p‐Erk activation in valve tissue. Furthermore, both refametinib and doxycycline attenuated elastolytic cathepsin K, L, MMP‐2, and MMP‐9 activation, and abrogated macrophage and neutrophil infiltration in *Emilin1* ^*−/−*^ aortic valves. RNAseq analysis was performed in aortic valve tissue from adult (4 months) and aged (14 months) *Emilin1* ^*−/−*^ and age‐matched wild‐type control mice, and demonstrated upregulation of genes associated with MAPK/MEK/p‐Erk signaling and elastases at the adult stage and inflammatory pathways at the aged stage controlling for age. These results suggest that Erk1/2 signaling is an important modulator of early elastase activation, and pharmacological inhibition using refametinib may be a promising treatment to halt AVD progression

## Introduction

Aortic valve disease (AVD) is a common cause of cardiovascular morbidity and mortality (Mozaffarian et al. 2015). Presently, there are no pharmacologic treatment options available for preventing, reversing, or halting the progression of AVD (Rajamannan et al. 2011). Therefore, surgery remains the primary treatment approach and this is restricted to severe “end stage” disease (Nishimura et al. 2014). Valve replacement procedures are associated with significant complications, and the need for reintervention is common (Gallegos 2006; Keane et al. 1993). Accordingly, there is a crucial need for new pharmacologic treatment options that stop AVD progression, precluding the need for surgical intervention. The National Heart, Lung, and Blood Institute has identified the need for new medical strategies applicable to early AVD (Rajamannan et al. 2011). Animal models that recapitulate the natural history of human AVD are required to optimally execute preclinical studies that test new therapeutic targets.

The *Emilin1* ^*−/−*^ mouse is a model of latent fibrotic AVD (Munjal et al. 2014). Emilin1 is an elastogenic glycoprotein that inhibits TGFβ‐mediated MEK/Erk1/2 signaling, and Emilin1 deficiency results in increased p‐Erk1/2 expression, elastase activation, and Vegf‐mediated aberrant angiogenesis in aortic valve tissue (Munjal et al. 2014). Interestingly, constitutively hyperactive Erk1/2 signaling results in valve maturation defects (Krenz et al. 2008). Importantly, the MAPK/p‐Erk1/2 pathway regulates the maladaptive response of valve interstitial cells (VICs), and inhibition of p‐Erk1/2 reduced this response in vitro (Gu and Masters 2009). Previous reports have shown a role for selective MEK1/2 inhibition in a mouse model of Marfan syndrome to treat thoracic aortic aneurysm (Holm et al. 2011), and MEK1/2 inhibitors mitigate pathological remodeling in mouse models of pulmonary fibrosis (Mercer and D'Armiento 2006). Several MEK1/2 inhibitors have successfully completed phase II clinical trial testing for various solid tumors (Schmieder et al. 2013). However, the potential in vivo therapeutic role of p‐Erk1/2 inhibition for AVD has not been tested.

Elastases are proteolytic enzymes that have the ability to cleave the elastic fibers resulting in elastic fiber fragmentation (EFF), a hallmark of AVD (Aikawa et al. 2009; Basalyga et al. 2004; Fondard et al. 2005; Schoen 1997; Vesely 1998). EFF, or elastase‐mediated elastic fiber assembly abnormalities, may contribute to AVD initiation and progression (Fondard et al. 2005; Hinton et al. 2006; Perrotta et al. 2011). Elastase inhibitors have been found to be successful in halting the progression of aortopathy and preventing aortic dissection (Xiong et al. 2012). Doxycycline, a nonspecific elastase inhibitor, is an FDA approved drug for elastolytic matrix metalloproteinase (MMP) inhibition in patients with periodontal disease (Gapski et al. 2009). Interestingly, one randomized clinical trial demonstrated that doxycycline had a pronounced effect mitigating inflammation in patients with aortopathy (Lindeman et al. 2009). Previous studies have suggested p‐Erk1/2 may be an important upstream regulator of elastase activation in aortic pathophysiology (Ghosh et al. 2012). However, the role of Erk1/2 signaling during AVD progression has not been demonstrated.

The goal of this study was to test three new pharmacologic treatment strategies for AVD in the *Emilin1*‐deficient mouse model, namely p‐Erk1/2 inhibition, elastase inhibition, and Vegf inhibition. We demonstrated the effectiveness of MEK/p‐Erk inhibition using refametinib, and to a lesser extent elastase inhibition using doxycycline. These findings have important clinical implications because understanding early disease mechanisms promises to identify new medical therapies. Refametinib with or without adjunct doxycycline warrants further investigation as a potential new medical therapy for AVD.

## Methods

### Study design

**Figure 1:**
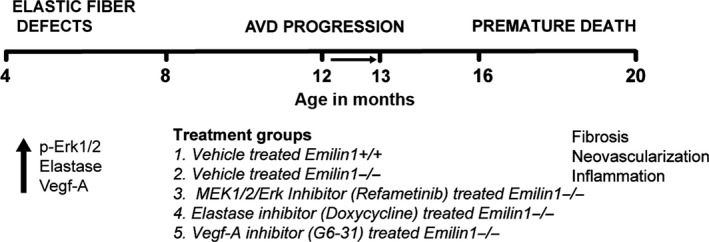
Preclinical study treatment strategy in Emilin1‐deficient mouse model of AVD. Emilin1 deficiency results in early p‐Erk1/2, elastase and Vegf activation at 4 month followed by the manifestation of AVD, characterized by fibrosis, neovascularization, and inflammation, at 12 months of age and premature death at 15–18 months of age. Treatment was initiated at 12 month age (arrow) and all five treatment groups were treated for 1 month period using drugs as mentioned.

*Emilin1* ^−/−^ and *Emilin1* ^*+/+*^ littermate mice were studied at 12 months of age. Mice were maintained on a C57Bl6 genetic background, and genotyping was performed as described previously (Munjal et al. 2014). Animals were divided into five groups: (1) vehicle‐treated *Emilin1* ^*+/+*^ mice (negative control); (2) vehicle‐treated *Emilin1* ^−/−^ mice (positive control); (3) *Emilin1* ^−/−^ mice treated with refametinib (RDEA‐119), a selective MEK1/2 inhibitor (p‐Erk1/2 inhibition); (4) *Emilin1* ^−/−^ mice treated with doxycycline (nonspecific elastase inhibition); and (5) *Emilin1* ^−/−^ mice treated with G6‐31 (Vegf‐A inhibition) (Fig. 1). All mice were treated for 4 weeks, including the vehicle control group. The Cincinnati Children's Research Foundation Institutional Animal Care and Use Committee approved all protocols.

Refametinib (RDEA‐119, BAY 869766), a MEK1/2 specific allosteric inhibitor (Chemical Genomics Center, NHGRI, National Institutes of Health), was administered twice daily using oral gavage at 25 mg/kg body weight per dose. RDEA‐119 was dissolved in DMSO at 10 mmol/L (Holm et al. 2011; Iverson et al. 2009; Wylie‐Sears et al. 2014) and reconstituted in 10% 2‐hydroxypropyl‐beta‐cyclodextrin (Sigma‐Aldrich, St. Louis). Doxycycline (0.5 g/L; Sigma) was delivered in drinking water based on established protocols (Chung et al. 2008; Xiong et al. 2008). Because doxycycline is light sensitive, water was shielded from all light and changed periodically every 2 days. G6‐31, an IgG1 monoclonal antibody that inhibits angiogenesis by neutralizing Vegf‐A (generously provided by Genentech Inc., San Francisco, CA), was given twice weekly for 4 weeks at a dose of 0.5 mg/kg by intraperitoneal injection. The concentrated drug was dissolved in 0.9% normal saline (Liang et al. 2006). The drug‐vehicle control group received 10% 2‐hydroxypropyl‐beta‐cyclodextrin, oral gavage, 0.9% normal saline, via an intraperitoneal route.

### Histology and immunohistochemistry

Mouse hearts were processed as previously described (Hinton et al. 2006; Munjal et al. 2014). Harts and Masson trichrome stains were used. Immunohistochemistry was performed to assess markers of p‐Erk1/2 signaling, elastase activation, angiogenesis, macrophage infiltration, and proliferation (Table S3). All antibody protocols used streptavidin/biotin colorimetry and diaminobenzidine detection. The slides were examined using Nikon NIS elements software. Immunostaining for Mmps and cathepsins were quantified using the National Institutes of Health (NIH) Image J software (http://rsb.info.nih.gov/ij/), as previously described (Jensen 2013).

### Western blotting

Analyses were performed on aortic valves (5 valves/experiment), as described previously (Munjal et al. 2014). A bicinchoninic acid protein assay kit (Pierce) was used to estimate total protein, and 30 μg of protein lysate was loaded onto 8–12% SDS–PAGE gels and then transferred onto nitrocellulose membranes. The membranes were blocked with 3% non‐fat dry milk in TBS‐T and incubated with primary for MMP‐2 and p‐Erk1/2 antibodies overnight at 4°C. Immunoblots were probed with HRP‐conjugated secondary antibodies for 1 h at room temperature and developed using chemiluminiscence (Amersham Biosciences). After stripping using the stripping buffer (Thermo Fischer), the blots were reprobed with Gapdh (Abcam) or t‐Erk1/2 antibody (Cell signaling). Signal intensity was quantified using the NIH Image J software. The arbitrary pixel densities of each protein were normalized to GAPDH or t‐Erk1/2. Band intensities on the western blots were quantified using Image Studio Lite software (Li‐COR).

### RNA‐Seq analysis

Aortic valves were dissected according to a protocol developed in the laboratory (Hinton et al. 2010; Munjal et al. 2014), pooled in RNALater (*n* = 10 mice/experiment) and stored according to manufacturer's recommendations. Total RNA was extracted using the micro RNEasy Kit (Qiagen, Valencia, CA), and RNA quality was assessed using the Agilent Bioanalyzer 2100. RNA was then prepared for sequencing using the Illumina mRNA‐seq Sample Prep Kit. The generated RNA‐seq libraries were subjected to high‐throughput 50 bp single‐end RNA‐sequencing at a read depth of >50 million reads utilizing the Illumina Hi‐Seq 2500 machine. All sequenced reads were aligned to the *mus musculus* (mm9 sequence database) subset of RefSeq using TopHat, and then processed with Cufflink to generate the transcriptome (Brunskill et al. 2014a,b; Potter and Brunskill 2014).

RNA‐Seq BAM files were imported into AvadisNGS software for further analysis. The RNA‐Seq data were then filtered for misaligned and/or duplicate reads. The filtered data was normalized using RPKM (reads per kilobase per million) and filtered again at a threshold of 10 RPKM. Differential expression analysis was performed on the filtered data set (>10 RPKM) to identify genes with a >2‐fold change.

In order to monitor the natural history of disease progression, the differentially expressed gene due to Emilin1 deficiency was monitored at early (4 month) and late (12–14 month) stages, corresponding with time points before and after disease onset, using enrichment analysis and compared with wild‐type control mice. Genes corresponding to differentially expressed transcript clusters were selected for display in hierarchical clustering, with a threshold *P*‐value cutoff of 0.05 using an one‐way ANOVA. Differentially expressed genes were clustered using Topp Cluster web server application for comprehensive analysis of multiple genes list or biological pathways in early and the late stage. The log2‐transformed data were preprocessed by median centering, and then hierarchically clustering method (https://toppcluster.cchmc.org/ ). Subsequently, enrichment maps and Venn diagrams were generated using cytoscape software (Barnette et al. 2014; Brunskill et al. 2014a,b; Potter and Brunskill 2014).

### Quantitative RT‐PCR

RNA from aortic valves (4 valves/experiment) was isolated from vehicle‐ and drug‐treated mice using Trizol. As previously reported, cDNA was generated using ~500 ng isolated RNA and amplified by PCR using gene‐specific primers (Table S4)(Hinton et al. 2010; Munjal et al. 2014). C_*t*_ values were obtained using Bio‐Rad software. The ΔΔC_*t*_ method was used to represent mRNA fold change. The experiments were performed in triplicate.

### Human valve tissue

Aortic valve specimens were obtained from nonsyndromic patients with isolated AVD and undergoing aortic valve replacement (affected), and from age‐matched individuals who died of noncardiac causes at the time of autopsy (control). AVD patients were stratified by age into early‐onset (0–40 years) and late‐onset (41–85 years) groups. Patients with a history of rheumatic heart disease or infective endocarditis were excluded. Control aortic valves from patients of similar ages were obtained at autopsy from individuals who died of noncardiac causes with a maximum ischemic time of 24 h. Aortic valve tissues were fixed in 10% formalin, dehydrated through a graded ethanol series, washed in xylenes, and embedded in paraffin wax and sectioned. Tissue slides were subsequently processed and stained as described previously (Wirrig et al. 2011). These studies were approved by the Institutional Review Boards at Cincinnati Children's Hospital Medical Center and Good Samaritan Hospital (Cincinnati, Ohio).

### Quantitative analysis of desmosine in human aortic valve tissue

Desmosine is an amino acid that resides within the crosslinking network of the mature elastic fiber. Desmosine quantitative assay is a marker for elastic fiber fragmentation that also reflects elastin content (Brown‐Augsburger et al. 1996). Frozen human aortic valve tissue isolated from patients undergoing valve replacement surgery was hydrolyzed in 6 N HCl at 100°C for 24 h, evaporated to dryness, and redissolved in water. The hydrolysates were evaporated to dryness, redissolved in water, and desmosine content was then measured as described previously (Starcher and Conrad 1995).

## Results

### Refametinib normalizes p‐Erk1/2 expression and attenuates EFF in *Emilin1* ^*−/−*^ aortic valve tissue

**Figure 2:**
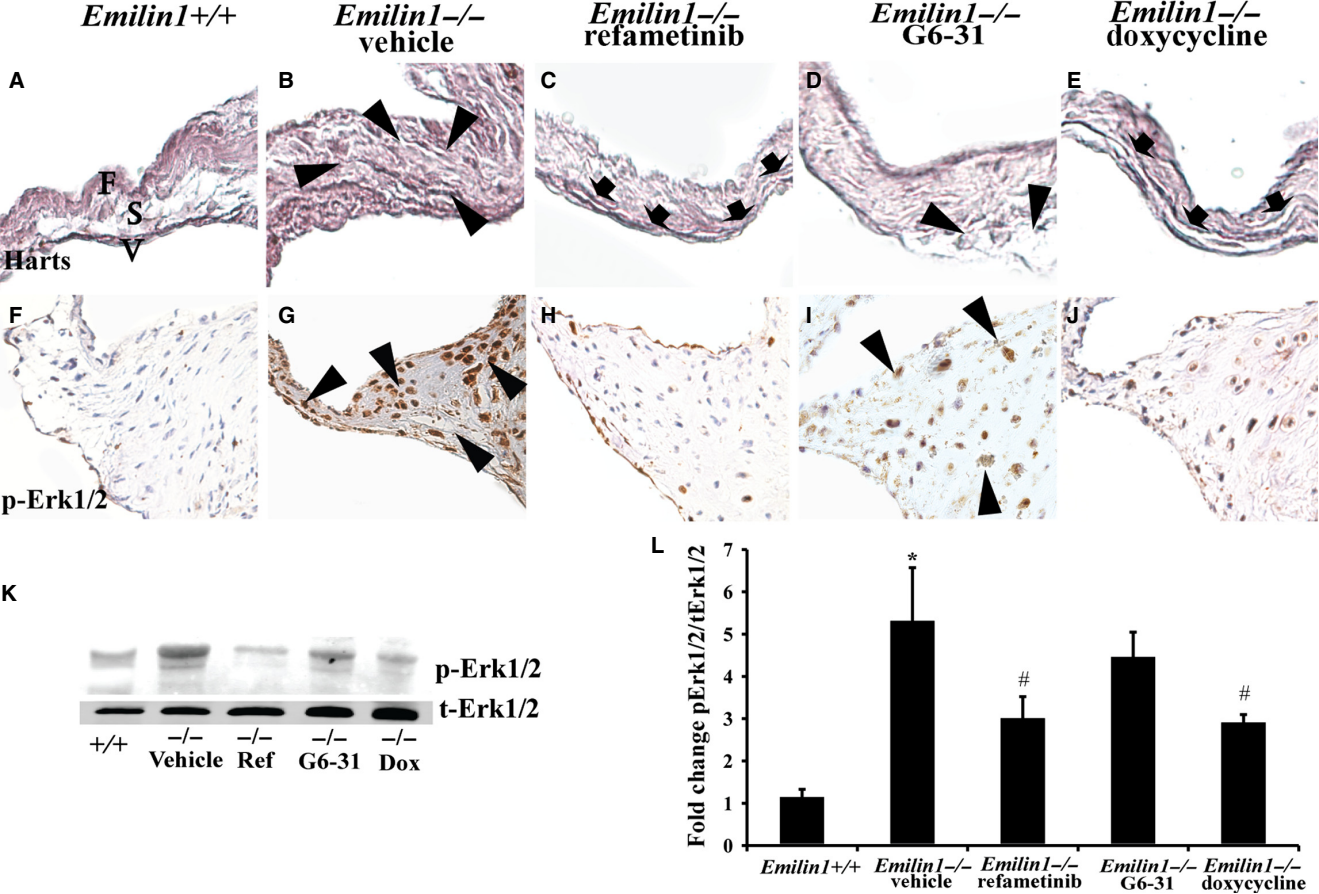
Refametinib, doxycycline, and G6‐31 treatment effects in Emilin1‐deficient mice. Harts micrograph showed elastic fiber assembly defects and EFF in *Emilin1‐/‐* aortic valves (arrowheads, B), and intact cusp layers, namely the fibrosa (F), spongiosa (S), and ventricularis (V) in *Emilin1* ^*+/+*^ valve (A), and reduction in EFF with refametinib (arrows, C) or doxycycline (arrows, E) treatment in aortic valve tissue. IHC shows p‐Erk1/2 activation in *Emilin1* ^*‐/‐*^ aortic valves (G) and dramatic reduction in p‐Erk1/2 expression in response to refametinib (H) or doxycycline treatment (J), and unaltered with G6‐31 (I) treatment. Western blot analysis (K) and corresponding densitometry analysis (L) show significant reduction in p‐Erk1/2 activation when treated with refametinib or doxycycline (n = 7–8/group, *P* < 0.0001; * different from *Emilin1* ^*+/+*^; # different from vehicle‐treated *Emilin1* ^*−/*−^). EFF, Elastic fiber fragmentation.

In aged *Emilin1* ^*−/−*^ mice with AVD characterized by EFF (Munjal et al. 2014), both refametinib and doxycycline, but not G6‐31, restored normal elastic fiber morphology (Fig. 2A–E). Refametinib demonstrated significant suppression of p‐Erk1/2 activation as evidenced by western blot and immunohistochemistry. Interestingly, doxycycline showed similar results. G6‐31 resulted in a mild reduction in p‐Erk1/2 expression that was not statistically significant (Fig. 2F–K). However, no change in neovessel formation or collagen deposition was seen in any treatment groups (data not shown). Densitometry evaluation further substantiated the findings showing significant reduction of p‐Erk1/2 to t‐Erk1/2 fold change in *Emilin1* ^*−/−*^ aortic valves when treated with refametinib or doxycycline (Fig. 2L). Taken together, these findings demonstrate that refametinib effectively suppresses p‐Erk1/2 in diseased tissue and restores elastic fiber morphology.

### Refametinib inhibits elastolytic enzymes in aortic valve tissue

**Figure 3:**
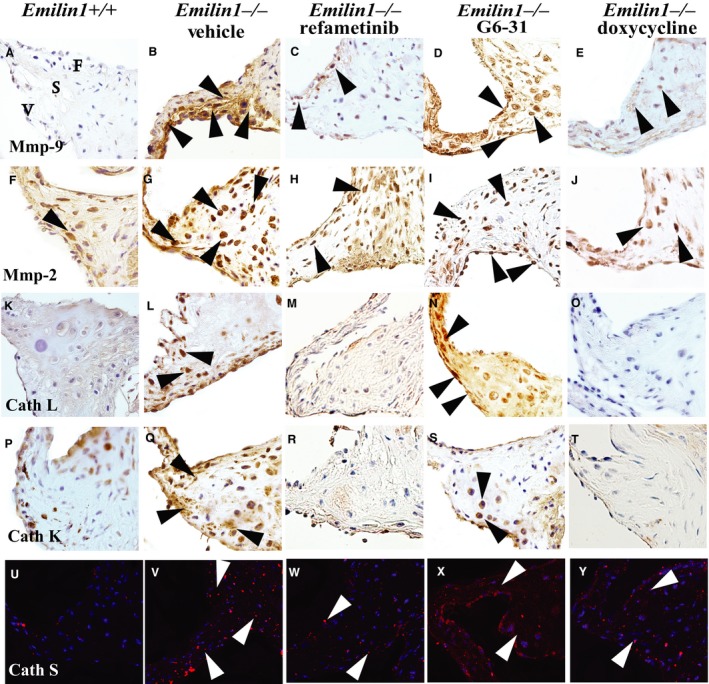
Refametinib and doxycycline mitigates pathological remodeling in *Emilin1* ^*−/−*^ aortic valve tissue. IHC showing Mmp‐9 (A–E), Mmp‐2 (F–J), cathepsin L (K–O), cathepsin K (P–T), and cathepsin S (U–Y) content and location. Mmp‐9 expression was increased in vehicle‐treated *Emilin1* ^*−/−*^ valves and was abrogated with doxycycline (D) or refametinib (E) treatment. Mmp‐2, which is expressed in *Emilin1* ^*−/−*^ aortic valves; on the other hand, it was unchanged with any treatment (H–J). Figure shows increased expression of cathepsin L (arrows, L) localized to the interstitial cells and cathepsin K (arrows, Q) localized to endothelial as well as interstitial cells in *Emilin1* ^*−/−*^ aortic valves, and this expression was dramatically reduced when treated with doxycycline (N, S) or refametinib (O,T). Cathepsin S expression was also increased in the interstitial cells of *Emilin1* ^*−/−*^ valves (V) but unchanged with any treatment.

*Emilin1* ^*−/−*^ aortic valves showed increased expression and activities of the elastolytic enzymes Mmp‐2 and Mmp‐9, indicative of pathological matrix remodeling due to Emilin1 deficiency. Interestingly, Mmp‐9 expression was suppressed with refametinib or doxycycline treatments (Fig. 3C, E). G6‐31 treatment, on the other hand, had no effect on Mmp‐2 or Mmp‐9 expression. We also examined cathepsins K, S and L in the extracellular matrix (ECM) remodeling established since studies have reported a role in for these enzymes in aortic valve disease (Helske et al. 2007). *Emilin1* ^*−/−*^ aortic valves demonstrate a robust increase in the expression of cathepsins L, K, and S in aged aortic valve tissue (Fig. 3L,Q,V). Doxycycline or refametinib treatment inhibits cathepsins K and L (Fig. 3M,R,O,T), consistent with previous studies showing either elastase or MEK1/2 inhibition reduces cathepsin K expression (Franco et al. 2011; Ihn et al. 2015). On the contrary, doxycycline or refametinib treatment did not effect cathepsin S expression. Overall, this suggests that p‐Erk1/2 inhibition using refametinib inhibits elastase activation and pathological matrix remodeling in *Emilin1* ^*−/−*^ aortic valves.

### Refametinib treatment resulted in a reduced inflammatory response but did not change aberrant angiogenesis

**Figure 4:**
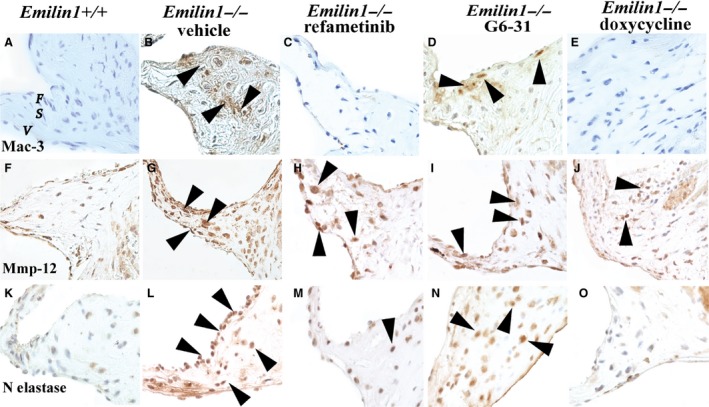
Refametinib and doxycycline abrogates macrophage infiltration in *Emilin1* ^*−/−*^ aortic valves. IHC shows Mac‐3 staining in *Emilin1* ^*−/−*^ aortic valve tissue that was completely obliterated with refametinib (C) or doxycycline (E), but not altered with G6‐31 treatment (D). Mmp‐12 was increased in hinge region of *Emilin1* ^*−/−*^ aortic valves (arrows, G) and was slightly decreased with doxycycline (J), but unaltered with refametinib (H) or G6‐31 (I) treatment. Neutrophil elastase was increased in aortic valve intestinal cells of *Emilin1* ^*−/−*^ mice (arrows, L), and this increase was dramatically decreased with refametinib (M) or doxycycline (O) but unchanged with G6‐31 (N).

Aged *Emilin1* ^*−/−*^ aortic valves manifest fibrosis, neovascularization, and inflammation (Munjal et al. 2014). To examine inflammation, IHC was performed using the macrophage marker Mac‐3, which identifies differentiated macrophages. *Emilin1* ^*−/−*^ valves showed significantly increased Mac‐3 when compared with control valves, and refametinib or doxycycline treatment, but not G6‐31 treatment, results in a dramatic reduction in Mac‐3 (Fig. 4). Mmp‐12 and neutrophil elastase (N‐elastase), nonspecific markers associated with inflammation, were both dramatically increased in *Emilin1* ^*−/−*^ valves, but Mmp‐12 and N‐elastase were reduced only slightly in all treatment groups. G6‐31‐treated *Emilin1* ^*−/−*^ valves showed reduction in Vegf‐A expression, however, refametinib‐ and doxycycline‐treated valves showed no change in Vegf‐A expression (data not shown). *Emilin1* ^*−/−*^ aortic valves are characterized by a progressive increase in the proliferative index and increased myofibroblast like phenotype in VIC's (Munjal et al. 2014), but neither the proliferation index nor the degree of myofibroblast interstitial cell activation was different with any treatment, as evidenced by p‐HisH3 and SM‐22 staining, respectively (data not shown). These findings suggest that refametinib abrogated macrophage and neutrophil infiltration in *Emilin1* ^*−/−*^ aortic valves, but did not halt the pathologic myoproliferative VIC response.

### RNAseq analysis demonstrated discrete early and late disease processes in Emilin1 deficient aortic valve tissue

In order to identify molecular signatures involved in AVD progression in *Emilin1* ^*−/−*^ mice, transcriptome analysis was performed at adult (4‐month‐old) and aged (14–18‐month‐old) stages in *Emilin1* ^*+/+*^ and *Emilin1* ^*−/−*^ aortic valves. RNAseq analyses revealed a total of 470 and 456 genes that were differentially expressed (twofold, *P *< 0.05) at adult and aged stages, respectively (Fig. S2). We studied whether the differentially expressed genes were enriched for specific signaling pathways. Genes involved in biological functions such as extracellular matrix disassembly, cartilage development, angiogenesis, and mechanical stimuli response elements were significantly enriched in *Emilin1* ^*−/−*^ adult aortic valves (Fig. S2). In addition, significant enrichment was found in genes involving TGFβ signaling in *Emilin1* ^*−/−*^ adults when compared with age‐matched controls. Aged *Emilin1* ^−/−^ aortic valves demonstrated significant enrichment in genes associated with inflammation. At least 17 genes were differentially expressed in both adult and aged *Emilin1* ^*−/−*^ mice, 4 are shown in the enrichment map (maroon nodes), suggesting different signaling pathways are involved in early and late disease processes (Fig. S2).

Interestingly, control wild‐type valves also demonstrate 281 differentially expressed genes due to aging. To validate the targets of altered differential gene expression in Emilin1 deficiency, additional IHC or QRT‐PCR was performed for selected genes **(**Fig. S3**)**. IHC revealed increased expression of the profibrotic gene *Periostin*, a marker for osteogenic procalcific activity gene *Spp‐1* (osteopontin) and the proinflammation gene *Ptx‐3* (Pentaxtrin) in *Emilin1‐/‐* aortic valves. Our RNAseq findings demonstrated elevated Ptx‐3 expression at both adult and aged stages in Emilin1‐deficient valves. Findings were corroborated using QRT‐PCR (Fig. S3). Furthermore, QRT‐PCR showed that Mmp‐2 expression was progressively increased in aged null valves when compared to wild‐type controls (Fig. S3). In addition, *Emilin1‐/‐* vascular smooth muscle cells (VSMCs) isolated from aortic root demonstrated a robust increase in p‐Erk1/2 expression when stimulated with the osteogenic media (OM). Neither aorta nor aortic valve demonstrated spontaneous gross calcification at any age in the *Emilin1* ^*−/−*^ mice (Fig. S4). VSMC's were isolated and cultured from the aortic root of *Emilin1* ^*+/+*^ *Emilin1* ^*−/−*^ mice. *Emilin1* ^*−/−*^ VSMCs calcify as seen by alizarin red staining when compared with *Emilin1* ^*+/+*^ cells. Osteocalcin and Runx‐2 (ossification markers) were upregulated in VSMC's cultured in OM (Fig. S4). Osteogenic media induces p‐Erk1/2 activation and downregulation of VEGF‐A, suggesting VSMC calcification is mediated by p‐Erk1/2 activation. Taken together, this suggests AVD pathogenesis in *Emilin1* ^*−/−*^ valves is dynamic, characterized by early perturbation in TGFβ signaling and ECM, followed by late inflammation and the manifestation of overt disease.

### Human AVD shows progressive p‐ERK1/2 and elastase activation

**Figure 5:**
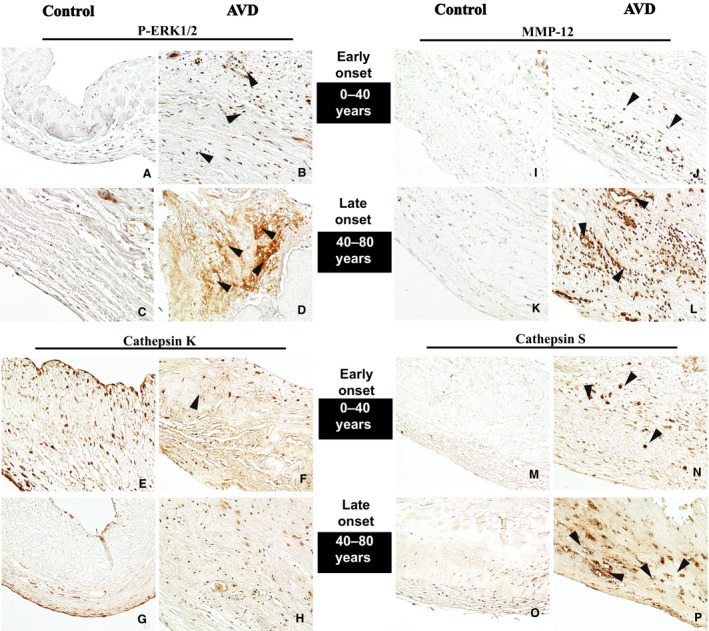
Human AVD tissue demonstrates similar findings as mouse model. Progressive p‐Erk1/2 activation in early (B) and late (D) onset AVD when compared to age‐matched WT controls. Cathepsin K was ubiquitously expressed in early controls (arrows, E, G) and early‐onset AVD (arrows, F, H). MMP‐12 and cathepsin S were increased in early‐onset AVD (arrows, J, N) compared to age‐matched controls, and further increased in late‐onset AVD (arrows, L, P) compared to early‐onset AVD.

To extend these observations to human tissue and determine the timing of potential p‐ERK1/2 activation in the context of human AVD, p‐ERK1/2 was examined using IHC. All human valves were processed within 6 h if not immediately. These studies demonstrated increased p‐ERK1/2 expression in early‐onset AVD in the absence of inflammation when compared with healthy control aortic valves, and p‐ERK1/2 expression was increased further in the presence of inflammation in late‐onset AVD (Fig. 5A–D). Cathepsin K was expressed ubiquitously in early‐onset AVD and age‐matched control aortic valve tissue, but continued to be expressed diffusely in late‐onset AVD while expression was restricted to the ventricularis layer in age‐matched controls (Fig. 5E–H). Cathepsin S is increased modestly in early‐onset AVD and increased dramatically in late‐onset AVD specimens when compared to age‐matched controls, which show no expression (Fig. 5M–P). Similarly, MMP‐12, a macrophage‐derived elastase, was increased slightly in early‐onset AVD but increased significantly in late‐onset AVD, including around neovessels (Fig. 5I–J). These histological images were quantified using Image J software, and comparisons across groups were performed as shown in Table S2. Taken together, these findings suggest that p‐ERK1/2 and elastolytic enzymes are misexpressed in early‐onset AVD, suggesting these early disease processes precede late inflammation in human AVD.

**Figure 6:**
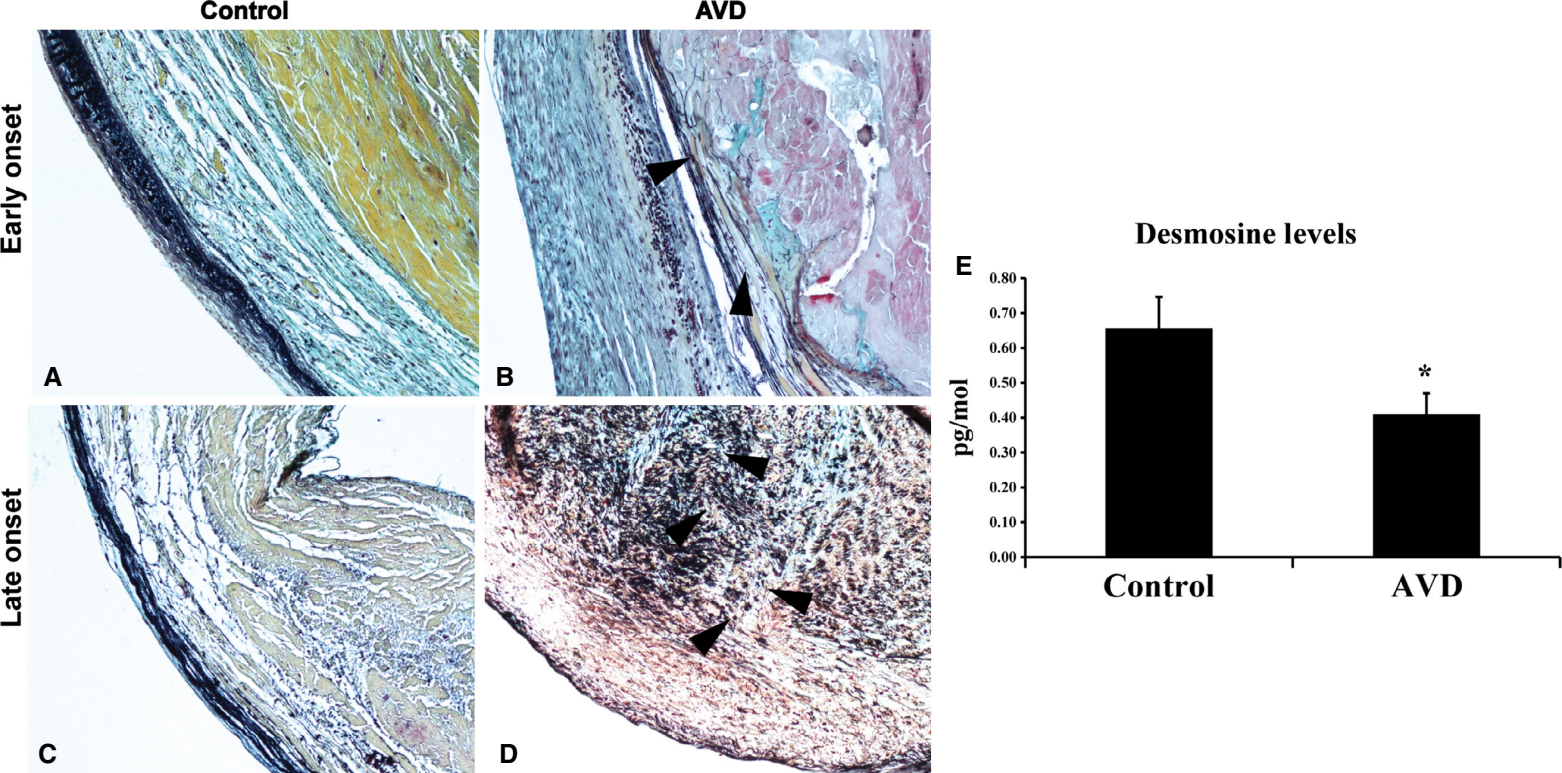
Human AVD tissue is characterized by early elastic fiber abnormalities. Normal trilaminar aortic valve architecture is seen in early (A) and late (C) controls. Elastic fiber fragmentation is evident in early‐onset AVD (B) and is worse in late‐onset AVD (D). There is a significant decrease in the degree of desmosine content in disease valves when compared to control valves (E). *n* = 8/group, *P* < 0.0001;*different from control.

To evaluate EFF in early and late‐onset AVD, histologic staining was performed, demonstrating marked elastic fiber defects in early‐onset AVD. In late‐onset AVD, there were similar elastic fiber abnormalities, including dispersion of fragments away from the ventricularis layer. Desmosine, a breakdown product of elastin as well as an index of elastic fiber turnover, was quantified in healthy and AVD specimens and showed significant reduction in AVD when compared to control valves (Fig. 6).

## Discussion

AVD is a burgeoning issue that effects more than 2% of the general population, causing more than 25,000 deaths annually in USA (Bonow et al. 2008; Nkomo et al. 2006). Clinical trials examining statins (HMG coenzyme reductase inhibitors) have shown that these drugs neither reverse AVD processes nor halt disease progression, e.g., the need for surgery and the timing of surgery is unchanged (Mohler et al. 2007). This is due in part to the relatively late initiation of therapy when tissue disease is advanced and inflammation and extensive calcification is already present, underscoring the importance of our need to understand early pathogenesis and develop strategies to begin therapy before these end stage milestones are realized. A NIH Working Group examining the state of research for AVD identified defining early intervention strategies as a priority and consequently the need for animal models that recapitulate the natural history of human AVD as well as molecular examination of the mechanisms involved in disease initiation and early progression. In the current study, we have evaluated the effectiveness of Erk1/2 inhibition, elastase inhibition and Vegf inhibition in reversing early disease processes in the *Emilin1*‐deficient mouse model of latent AVD (Munjal et al. 2014). Our results suggest Erk1/2 inhibition using refametinib and to a lesser extent elastase inhibition using doxycycline are new potential therapeutic targets.

**Figure 7:**
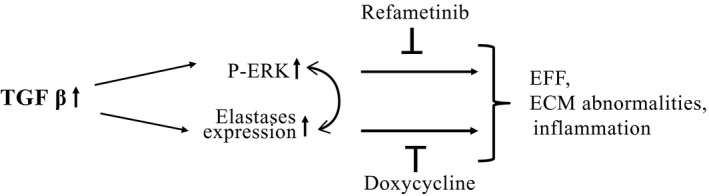
Summary model of proposed mechanism of AVD progression. TGFβ signaling results in Erk1/2 activation and this in turn stimulates elastase expression ultimately leading to the downstream effects including ECM abnormalities and inflammation. Refametinib or doxycycline treatment, inhibiting p‐Erk1/2 or elastase activation, significantly reverses progression of disease; however, the fact that neither drug returns p‐Erk to its WT levels suggests that Erk1/2 phosphorylation and elastase expression potentiate reciprocally. AVD, Aortic valve disease.

The results of the current study demonstrate that increased noncanonical TGFβ signaling in *Emilin1* ^*−/−*^ aortic valves induces increased expression of both p‐Erk1/2 and elastases that eventually results in EFF and inflammation. Refametinib or doxycycline treatment, inhibits p‐Erk1/2 or elastase expression; however, the fact that neither drug returns p‐Erk1/2 expression to its wild‐type levels suggests that p‐Erk1/2 and elastase expression potentiate reciprocally (Fig. 7). In this model, it is unclear whether TGFβ activation is canonical or noncanonical. TGFβ signaling results in Erk1/2 activation and this in turn stimulates elastase expression ultimately leading to the downstream effects including ECM abnormalities and inflammation. However, we show that doxycycline not only blocks downstream targets but also Erk1/2 phosphorylation (Fig. 2K and L), suggesting reversible elastases and Erk1/2. Therefore, complex interactions between TGFβ targets are present in aortic valve tissue in the *Emilin1* ^*−/−*^ mouse model of latent AVD.

Previous studies have established aging as the major risk factor for developing aortic valve disease (Rossebo and Pedersen 2004). Our transcriptome analysis indicated altered genes associated with ECM turnover and organization at the adult stage and activation of genes involved in inflammation at the aged stage in Emilin1‐deficient aortic valves. At least 17 genes were differentially expressed in both adult and aged *Emilin1* ^*−/−*^ mice (Fig. S2), 4 are shown in the enrichment map (maroon nodes), suggesting different signaling pathways are involved in early and late disease processes (Fig. S1).

Interestingly, control wild‐type valve tissue also depicted altered gene expression related to inflammation, albeit only a lesser degree, due to aging, suggesting the mutant mouse may show advanced aging, consistent with clinical studies identifying age‐related inflammation as an independent risk factor for AVD. The Emilin1‐mutant valve does not only grossly calcify at any age, including 24 months (unpublished), but also there are multiple pieces of data that suggest elevated osteopontin is present in *Emilin1* ^*−/−*^ valve tissue. Moreover, recent studies suggest that osteopontin has a role as an angiogenic factor, in addition to its role as a marker for osteogenic procalcific activity, and also our previous studies have shown elevated level of proangiogenic factors in *Emilin1* ^*−/−*^ valves (Poggio et al. 2014). In addition, our studies have shown vascular smooth muscle cells isolated from aortic root of *Emilin1* ^*−/−*^ spontaneously calcify when stimulated with osteogenic media. Also, osteopontin expression was increased in both cell types in the presence of osteogenic media. Taken together, aging may exacerbate AVD progression in genotype‐predisposed (*Emilin1* ^*−/−*^) valve tissue, recapitulating human AVD pathogenesis that initiates with perturbations in ECM remodeling and progresses to advanced disease with inflammation.

The findings of the current study are relevant to human disease. Human AVD is characterized by a vast array of pathologic findings, and discerning primary and secondary findings is critical to understanding natural history and identifying new therapeutic targets. In the current study, our human data suggest that P‐ERK1/2 was localized more in the nuclei in the early‐onset disease valves in contrast with late‐onset disease that showed more pronounced expression in the matrix. We do not have any evidence that these treatments rescued valve fibrosis. Grossly, pentachrome staining did not demonstrate a difference in collagen content with treatment and quantitatively, QRT‐PCR did not normalize fibrillar collagens. The expression of proteolytic enzymes declined in Emilin1‐deficient valves when treated with refametinib, suggesting a therapeutic role of MEK inhibition in mitigating pathological ECM remodeling and this might be extrapolated to suggest that the progressive nature of fibrosis would discontinue but the existing fibrosis would be unchanged. We also showed a marked increase in Cathepsin S expression in late‐onset human AVD, that is AVD associated with inflammation. Combined with observations in the *Emilin1* ^*−/−*^ mouse valves that showed increased expression of elastolytic enzymes (Mmp‐2,9, and cathepsins K, L, and S), this suggests that early elastase activation may be related to elastic fiber abnormalities while late elastase activation may be secondary to inflammatory processes and these patterns may be distinct. Importantly, refametinib or doxycycline treatment significantly decreased the expression of proteolytic enzymes Mmp‐9, and cathepsin K and L. Elastic fiber degradation products have shown promise as biomarkers of emerging disease states (Marshall et al. 2013). Previous studies have shown that inhibition of MEK/2 is a potential therapeutic option for valve disease patients (Sauls et al. 2015). Taken together, this suggests elastases may be potential markers for early AVD pathogenesis, consistent with our hypothesis that elastic fiber abnormalities play a fundamental role in AVD initiation and progression prior to inflammation.

This study has significant limitations. One limitation is the lack of data examining the effects of combined therapy using refametinib and doxycycline, which hypothetically may have an additive or synergistic effect, and in the event p‐Erk1/2 upregulation were completely rescued, evidence that both Erk and elastases potentiate reciprocally. Interestingly, myofibroblast cell activation and proliferation, which is markedly increased in *Emilin1* ^*−/−*^ aortic valves, was not reduced with either treatment, suggesting a VIC myofibroblast‐like switch occurred prior to treatment, consistent with the idea that ECM accumulation and disorganization is an early disease process. It did not alter myofibroblast activation, angiogenesis or fibrosis. However, the current study has identified early disease processes involved in progression that will provide a basis to consider new therapies for early‐onset AVD. In our previous study (Munjal et al. 2014), we showed a significant increase in the average velocity across the mutant aortic valve, but some aged valves had pathologic changes without physiologic changes (i.e., normal velocity) so only a proportion of mutant valves had increases that would be considered consistent with clinically significant human AVD. We interpret these findings to be an age‐related phenomenon in part because we have followed mice to 24 months at which time they are more likely to have increased velocities. Additional support of valve pathology is the coexistence of aortic insufficiency in some mice, a finding that is abnormal and often observed in the context of a malformed or diseased valve. Taken together, the data demonstrate a beneficial pathological response to MEK/Erk inhibition and elastase inhibition, but it is unclear at present if this extends to physiologic rescue in the subset that has disease.

## Author Contributions

C.M., G.M.B, and R.B.H. conceived and designed the study; C.M., A.G.J, A.M.O., R.A.N., C.J.T., J.M.S., and R.P.M. acquired the data for the study; C.M., A.G.J., A.M.O., R.P.M., G.M.B., and R.B.H. analyzed and interpreted the data; C.M and R.B.H. drafted the manuscript; C.M., A.G.J., A.M.O., R.A.N., C.J.T., J.M.S., R.P.M., G.M.B., and R.B.H. carried out critical revision and final approval of the manuscript.

## Conflict of Interest

None declared.

## Data Accessibility

## Supporting information




**Table S1**. Staining quantification of mouse histology.
**Table S2**. Staining quantification of human histology.
**Table S3**. Specification of the primary antibodies.
**Table S4**. Specification of the primers.
