# Supplementary material for: Inhibition of MAPK‐Erk pathway in vivo attenuates aortic valve disease processes in Emilin1‐deficient mouse model
Source: Physiol Rep. 2017 Mar 7;5(5):e13152. doi: 10.14814/phy2.13152 (PMC5350168; doi:10.14814/phy2.13152)
Supplement: Supplementary file 1 — Table S1. Staining quantification of mouse histology. Table S2. Staining quantification of human histology. Table S3. Specification of the primary antibodies. Table S4. Specification of the primers. [file PHY2-5-e13152-s001.docx]

# Supplemental tables

# **Inhibition of MAPK-Erk pathway *in vivo* attenuates aortic valve disease processes in Emilin1 deficient mouse model**

| **Supplemental Table 1. Staining quantification of mouse histology** | | | | | |
| --- | --- | --- | --- | --- | --- |
|  | *Emilin1+/+* | *Emilin1-/-* Vehicle | *Emilin1-/-* Refamatinib | *Emilin1-/-*  G6-31 | *Emilin1-/-* Doxycycline |
|  | Mean±SEM (A.U) | Mean±SEM (A.U) | Mean±SEM (A.U) | Mean±SEM (A.U) | Mean±SEM (A.U) |
| Mmp-2 | 0.022±0.03 | 0.079±0.006* | 0.023±0.001# | 0.07±0.008 | 0.028±0.006# |
| Mmp-9 | 0.014±0.001 | 0.089±0.005* | 0.025±0.013# | 0.078±0.003 | 0.047±0.006# |
| Mmp-12 | 0.021±0.003 | 0.068±0.008* | 0.036±0.015 | 0.065±0.003 | 0.044±0.02 |
| Cathepsin K | 0.013±0.001 | 0.064±0.011* | 0.032±0.011 | 0.079±0.042 | 0.034±0.009 |
| Cathepsin L | 0.014±0.001 | 0.064±0.003* | 0.066±0.012 | 0.066±0.012 | 0.038±0.004 |

| **Supplemental Table 2. Staining quantification of human histology** | | | | |
| --- | --- | --- | --- | --- |
|  | Early Control | Late Control | Early Disease | Late Disease |
|  | Mean±SEM (A.U) | Mean±SEM (A.U) | Mean±SEM (A.U) | Mean±SEM (A.U) |
| P-ERK | 0.009±0.002 | 0.014±0.002 | 0.051±0.015* | 0.07±0.009# |
| CATHEPSIN K | 0.038±0.06 | 0.054±0.009 | 0.066±0.011 | 0.047±0.003 |
| CATHEPSIN S | 0.019±0.006 | 0.023±0.002 | 0.034±0.007* | 0.075±0.015# |
| MMP-12 | 0.005±0.002 | 0.01±0.003 | 0.017±0.005* | 0.034±0.016 |

| **Supplemental Table 3. Specification of the primary antibodies** | | | |
| --- | --- | --- | --- |
| **Protein** | **Marker** | **Host and Type** | **Source** |
| Neutrophil elastase | Elastase | Goat polyclonal | Santa Cruz |
| Mmp-2 | Non-specific Elastase | Rabbit polyclonal | Abcam |
| Mmp-9 | Non-specific Elastase | Rabbit polyclonal | Abcam |
| Mmp-12 | Macrophage, Elastase | Rabbit polyclonal | Abcam |
| Cathepsin K | Elastase | Rabbit polyclonal | Abcam |
| Cathepsin L | Elastase | Mouse monoclonal | Abcam |
| Cathepsin S | Elastase | Goat polyclonal | Abcam |
| p-Erk1/2 | Map kinase | Rabbit polyclonal | *Invitrogen* |
| t-Erk1/2 | Map kinase | Rabbit polyclonal | Millipore |
| Cleaved Caspase | Apoptosis | Rabbit polyclonal | Cell signaling |
| Ki67 | Proliferation | Rabbit polyclonal | Abcam |
| Mac-3 | Macrophage | Rabbit polyclonal | Abcam |
| Pentaxtrin | RNAseq Validation | Rabbit polyclonal | Abcam |
| Periostin | RNAseq Validation | Rabbit polyclonal | Abcam |

| **Supplemental Table 4. Specification of the Primers** | | | |
| --- | --- | --- | --- |
| Gene | Annealing Temp (^0^C) |  | Primer Sequences |
| mPtx-3 | 55 | F | CGT GCG TGG TTG GTT CAA GAA C |
|  |  | R | GCG GTG AGA ATA CAG GTT GTG AA |
| mMmp-2 | 55 | F | AAC TAC GAT GAT GAC CGG AAG TG |
|  |  | R | TGG CAT GGC CGA ACT CA |
| mSpp1 | 58 | F | GAT GCC ACA GAT GAG GAC CTC |
|  |  | R | CTG GGC AAC AGG GAT GAC AT |
| mPTN | 55 | F | AAT GCT GCC CTG GCT ATA TG |
|  |  | R | GTA GTG GCT CCC ACA ATG C |
| mGAPDH | 55 | F | GCA CAG TCA AGG CCG AGA AT |
|  |  | R | GCC TTC TCC ATG GTG GTG AA |
